# Heat shock transcription factor A1b regulates heat tolerance in wheat and Arabidopsis through OPR3 and jasmonate signalling pathway

**DOI:** 10.1111/pbi.13268

**Published:** 2019-11-06

**Authors:** Xuejun Tian, Fei Wang, Yue Zhao, Tianyu Lan, Kuohai Yu, Liyuan Zhang, Zhen Qin, Zhaorong Hu, Yingyin Yao, Zhongfu Ni, Qixin Sun, Vincenzo Rossi, Huiru Peng, Mingming Xin

**Affiliations:** ^1^ State Key Laboratory for Agrobiotechnology Key Laboratory of Crop Heterosis and Utilization (MOE) Key Laboratory of Crop Genomics and Genetic Improvement (MOA) Beijing Key Laboratory of Crop Genetic Improvement China Agricultural University Beijing China; ^2^ Council for Agricultural Research and Economics Research Centre for Cereal and Industrial Crops Bergamo Italy

**Keywords:** wheat, heat stress, *TaOPR3*, *HSFA1b*, *DREB2A*

## Competing interests

The authors declare no competing interests.

## Author contributions

M.X. and H.P. designed the experiments. X.T., F.W., Y.Z., T.L., K.Y., L.Z. and Z.Q. performed the research. Z.H., Y.Y. and Z.N. analysed the data. Q.S. V.R. H.P. and M.X. wrote the paper.

Dear Editor,

High temperature adversely affects plant growth and severely causes crop yield loss worldwide, especially for chimonophilous wheat (*Triticum aestivum* L.; Akter and Islam, 2017). Heat shock transcription factors (HSFs) and plant hormones play regulatory roles in plant responses to heat stress (Baniwal *et al*., 2004). In this study, we found that *TaOPR3* contributes to heat tolerance in wheat probably via regulating JA level.

**Figure 1:**
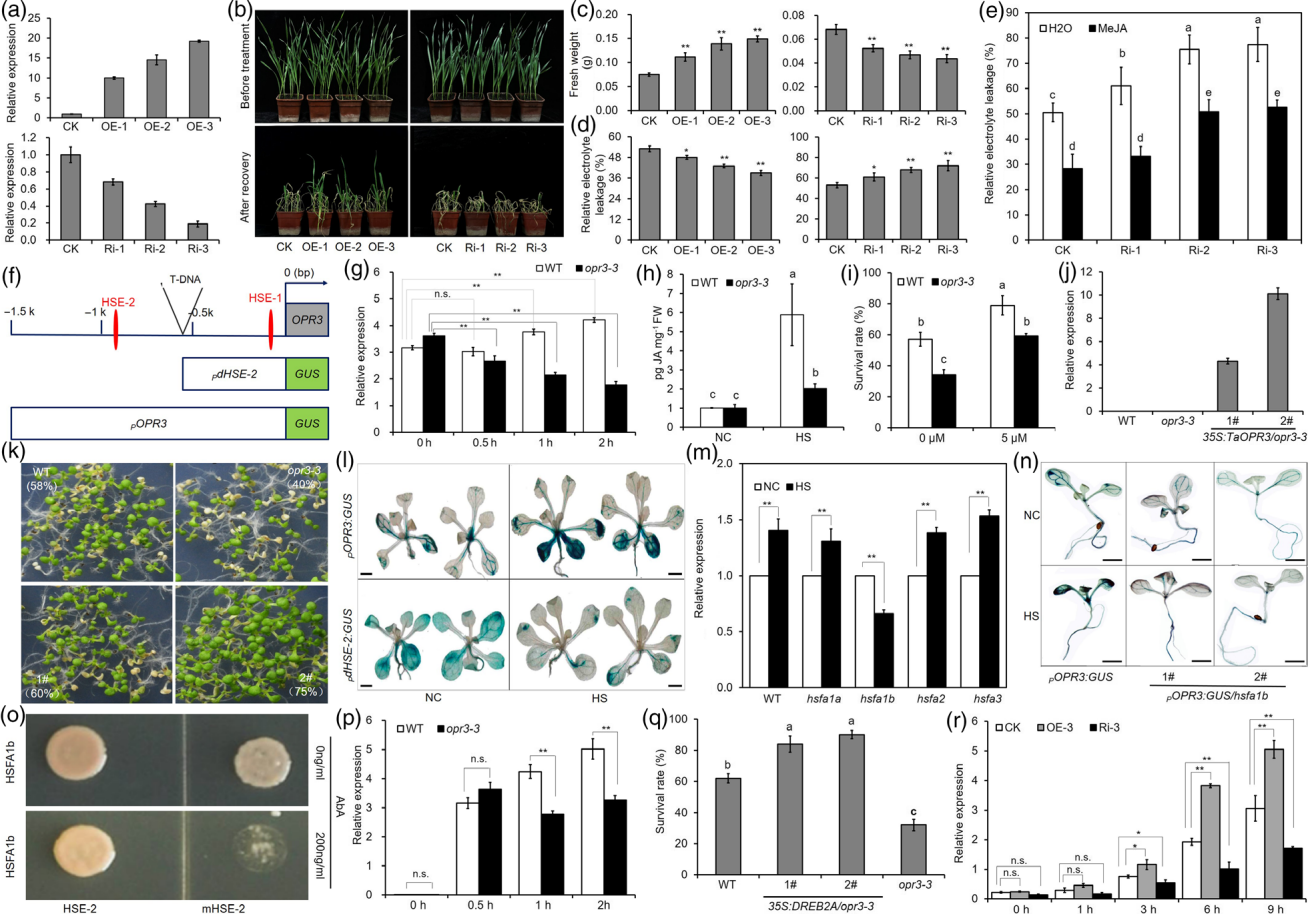
OPR3 contributes to heat tolerance in wheat and Arabidopsis (a) Expression analysis of *TaOPR3* in KN199 (CK), *TaOPR3* RNAi and overexpression transgenic lines. (b) Phenotypic analysis of KN199, *TaOPR3* RNAi and overexpression transgenic lines in response to heat stress. Statistical analysis of fresh weight (c) and electronic leakage (d) for CK, Ri and OE seedlings after heat stress. (e) Statistical analysis of electronic leakage of CK, Ri and OE seedlings with or without MeJA treatment after heat stress. (f) Schematic diagram of T‐DNA insertion and HSE elements in *AtOPR3* gene in *opr3‐3* mutant as well as GUS reporter construction. (g) Expression analysis of *AtOPR3* in response to heat stress in WT and *opr3‐3* by qRT‐PCR. (h) Endogenous JA levels in WT and *opr3‐3* seedlings before (NC) and after (HS) heat treatment. (i) Statistic analysis of survival rate for WT and *opr3‐3* seedlings with or without MeJA treatment after heat treatment. (j) Expression analysis of *TaOPR3* in WT,* opr3‐3* and *35S:TaOPR3/opr3‐3* by qRT‐PCR. (k) Heat tolerance analysis of WT,* opr3‐3* and *35S:TaOPR3/opr3‐3* seedlings. (l) GUS staining analysis of *pOPR3:GUS*/WT and *pdHSE2:GUS*/WT seedlings before (NC) and after (HS) heat treatment. (m) Expression analysis of *AtOPR3* in *hsfa1a*,* hsfa1b*,* hsfa2* and *hsfa3* mutants before (NC) and after (HS) heat stress by qRT‐PCR. (n) GUS staining analysis of *pOPR3:GUS*/WT and *pOPR3:GUS*/*hsfa1b* before (NC) and after (HS) heat treatment. (o) Yeast one‐hybrid detects HSFA1b and HSE‐2 interaction. (p) Expression analysis of *DREB2A* in response to heat stress in WT and *opr3‐3* by qRT‐PCR. (q) Statistic analysis of survival rate for WT,* opr3‐3* and *35S:DREB2A/opr3‐3* seedlings after heat treatment. Each experiment was performed in triplicate. (r) Expression analysis of *TaDREB2A* in KN199, *TaOPR3* RNAi and overexpression transgenic lines in response to heat stress. Data represent mean ± SD. n.s. indicates *P* > 0.05, * indicates *P* < 0.05 and ** indicates *P* < 0.01 (student's *t*‐test). Letters indicate significant differences (*P* < 0.05, LSD test). Scale bars = 1 mm.

To investigate the biological function of *TaOPR3* in heat responses, we generated *TaOPR3* RNAi (Ri1‐3) and overexpression (OE1‐3) lines (Figure 1a). Under normal conditions, no obvious phenotypic variation was detected between *TaOPR3* transgenic lines and ‘KN199’ (CK) (Figure 1b). However, under heat stress conditions, Ri and OE lines were more sensitive and resistant to heat stress than CK, respectively (Figure 1b). Specifically, Ri and OE lines exhibited a reduction (0.047 g vs. 0.068 g, on average) and increase (0.133 g vs. 0.075 g, on average) of fresh weight, respectively (Figure 1c). In addition, electrolyte leakage level was higher (67%) in Ri lines and lower (43%) in OE lines compared with CK (53%) (Figure 1d). Furthermore, the heat‐sensitive phenotype, in terms of electrolyte leakage level, of Ri lines was reduced by exogenous application of MeJA (from an average of 33% to 61%; Figure 1e).

To better understand how plants respond to elevated temperatures via JA pathway, we identified a new T‐DNA insertion mutant allele of *AtOPR3* in Arabidopsis (SALK_053805; hereafter named as *opr3‐3*) (Figure 1f). Unlike previously reported *opr3* mutant alleles (Chehab *et al*., 2011), *AtOPR3* expression is slightly increased under normal conditions but no obvious phenotypic variation is observed in *opr3‐3*. However, the transcript level of *AtOPR3* is decreased in *opr3‐3* after heat treatment (Figure 1g). Accordingly, a reduction in JA level to heat stress was detected in *opr3‐3* compared to WT (Figure 1h). The *opr3‐3* plants were more sensitive to heat stress than WT (Figure 1i). Nevertheless, the heat‐sensitive phenotype of *opr3‐3* was rescued by exogenous application of MeJA (Figure 1i). In addition, overexpression of wheat *TaOPR3* re‐establishes heat tolerance in Arabidopsis *opr3‐3* mutant, suggesting that OPR3‐mediated thermotolerance may be conserved between Arabidopsis and wheat (Figure 1j and k).

To elucidate the underlying mechanism responsible for the transcriptional regulation of *AtOPR3* under heat stress conditions, the putative *AtOPR3* promoter sequence (1500 bp) was analysed using Plant CARE interface programme. Two potential heat shock elements (HSE‐1 and HSE‐2) at the position of ‐175 bp and ‐903 bp were identified (Figure 1f). Interestingly, the *opr3‐3* T‐DNA insertion is located between the two HSEs. Next, *GUS* reporter was fused with integral 1500‐bp promoter sequence (*pOPR3:GUS*; including both HSE‐1 and HSE‐2) and with a 620 bp sequence only including HSE‐1 but not HSE‐2 (*pdHSE‐2:GUS*) (Figure 1f). Results showed that deletion of HSE‐2 affects GUS expression in response to heat stress (Figure 1l).

HSEs are usually bound by HSFs to regulate gene expression (Wu, 1995). Among four *hsf* mutants (*hsfa1a*,* hsfa1b*,* hsfa2* and *hsfa3*), we found that *AtOPR3* transcript abundance is reduced only in *hsfa1b* mutant after heat treatment (Figure 1m). Next, we introduced *pOPR3:GUS* construct into both WT and *hsfa1b* mutant and found that *pOPR3:GUS*/WT lines exhibited up‐regulated GUS expression level after heat treatment, but not for *pOPR3:GUS*/*hsfa1b* lines (Figure 1n). Yeast one‐hybrid assay indicated that yeast strain co‐transformed with vector expressing HSFA1b plus vector containing canonic HSE‐2 sequence grow on selective media (media lacking leucine and containing 200 ng/ml AbA), while strain co‐transformed with mHSE‐2 (a substitute of HSE‐2) is unable to grow (Figure 1o).

To further shed light on the mechanisms linking JA signalling pathway with heat stress tolerance, we investigated the expression pattern of JA inducible gene *DREB2A* and found that *DREB2A* mRNA level is lower in *opr3‐3* than in WT after 1 h and 2 h heat treatment, (Figure 1p). Constitutively expressing *DREB2A* in *opr3‐3* mutants (*35S:DREB2A/opr3‐3*) exhibited enhanced heat tolerance with higher survival rate (84%‐90%) than that of *opr3‐3* (31%) and WT (62%) after heat treatment (Figure 1q). These results suggest that JA affects heat tolerance by regulating *DREB2A*. We also found that the expression level of *TaDREB2A* is impaired in *TaOPR3* RNAi lines compared with WT at 3 h, 6 h and 9 h after heat stress, whereas it is enhanced in *TaOPR3* overexpression lines (Figure 1r), further indicating a potentially similar mechanism of heat stress tolerance in wheat and Arabidopsis.

Limited information is available about molecular mechanisms of the JA‐mediated thermotolerance. In present study, we provide information which improves knowledge regarding the mechanistic link between heat stress/HSFs and JA signalling pathway. When plants perceive heat stress, HsfA1b might convert into a functional homo‐trimer (Peteranderl *et al*., 1999; Wu, 1995) and activates *AtOPR3* expression. This leads to increased JA biosynthesis and accumulation. Subsequently, JA‐mediated signalling pathway activates a cascade resulting in increased *DREB2A* expression and enhanced heat tolerance in plants. Our study provides a potential approach to improve crop heat stress tolerance by increasing *OPR3* expression level appropriately under heat stress conditions.
